# Study on the Functional, Physiological, and Sensory Properties of Coffee Leaf Dark Tea Processed Using the Pile Fermentation Method of Pu-Erh Tea

**DOI:** 10.3390/foods15111980

**Published:** 2026-06-03

**Authors:** Yanbing Wang, Kun Zhang, Guosheng Xiong, Ping Du, Jianhua Dong, Jinxue Li, Xiaogang Liu, Zhenjia Chen

**Affiliations:** 1 Faculty of Modern Agricultural Engineering, Kunming University of Science and Technology, Kunming 650500, China; wongyb@126.com (Y.W.);; 2 Research Center for Analysis and Measurement, Kunming University of Science, Kunming 652094, China; 3 Analytic & Testing Research Center of Yunnan, Kunming 652094, China; 4 College of Biotechnology and Engineering, West Yunnan University, Lincang 677000, China; 5 College of Agronomy and Biotechnology, Dehong Normal University, Mangshi 678400, China; 6 China Coffee Engineering Research Center, Mangshi 678400, China

**Keywords:** coffee leaf dark tea, Pu-erh-style pile fermentation, microbial inoculation, antioxidant, metabolic activities, sensory properties

## Abstract

Coffee leaves are a promising raw material for functional beverages due to their distinctive phytochemical profile. In this study, sun-dried Arabica coffee leaves were processed into coffee leaf dark tea using Pu-erh-style pile fermentation with three treatments: spontaneous pile fermentation (SPPF), *Saccharomyces cerevisiae*-inoculated fermentation (SIPF) and *Aspergillus niger*-inoculated fermentation (AIPF). Changes in basic components, tea pigments, key phytochemicals, antioxidant capacity, in vitro hypoglycemic and hypolipidemic activities, and sensory properties were evaluated over 25 days. Pile fermentation reduced water extract, free amino acids and several phenolic constituents, while promoting the formation of theabrownins, particularly in SIPF and AIPF. Total phenolics, flavonoids, and antioxidant activities increased initially and then declined, with higher bioactivities observed at intermediate fermentation times. Coffee leaf dark tea also exhibited in vitro α-amylase and α-glucosidase inhibition, glucose diffusion retardation, reduced starch digestibility, and pancreatic lipase inhibition, with SIPF and AIPF outperforming SPPF. Sensory evaluation showed that inoculated fermentations, especially AIPF at 15 days and SIPF at 10 days, produced teas with superior overall quality. These results suggest that Pu-erh-style pile fermentation with targeted microbial inoculation may be a feasible strategy to obtain coffee leaf dark tea with enhanced in vitro functional-related properties and desirable sensory characteristics.

## 1. Introduction

Global coffee production reached 11 million tons of green beans in 2023, making it a key crop that supports tropical agriculture, international trade, and livelihoods worldwide [1,2]. However, large-scale coffee production results in a significant number of agricultural by-products, including coffee husks, pulp, silverskin, spent coffee grounds, and leaves, which are rich in phytotoxic and/or antinutrient compounds [3,4]. As coffee consumption rises globally, concerns over the environmental impact and resource efficiency of coffee by-products have emerged [5]. In response, research has increasingly focused on finding ways to utilize these by-products as a source of bioactive compounds for applications in food, cosmetics, and pharmaceuticals [3]. Among them, coffee leaves are attracting interest for their potential in creating health-promoting beverages [6].

Coffee leaves contain bioactive compounds like caffeine, trigonelline, chlorogenic acids, mangiferin, and rutin, which are known for their strong antioxidant, anti-inflammatory, and metabolic regulatory effects [7,8]. Unlike coffee beans, which require roasting and extraction before consumption, coffee leaves can be processed directly into infusions, making them a more sustainable and convenient resource. Due to the biochemical similarities between coffee leaves and tea leaves (*Camellia sinensis*), Chinese researchers have explored the application of traditional tea-processing techniques to coffee leaves, including withering, fixation, rolling, and fermentation [9,10]. These studies suggest that tea-style processing can enhance the sensory attributes of coffee leaves and modify their phytochemical composition, potentially improving their physiological activity.

Tea, a traditional beverage with a history spanning thousands of years in China, is categorized by fermentation level into green (0%), white (5–10%), yellow (10–20%), oolong (15–70%), black (70–90%), and dark tea (post-fermentation) [11]. Among these, dark tea is the most distinctive, as it undergoes a distinctive post-fermentation process driven by extraneous microbial activity [11,12]. Yunnan Pu-erh tea, one of the most famous dark teas, undergoes a pile fermentation process primarily driven by *Aspergillus niger*, assisted by yeast and bacteria. Pu-erh-style pile fermentation is a controlled post-fermentation process in which moistened tea leaves are piled under warm and humid conditions to promote microbial growth and biochemical transformation. During this process, microorganisms such as fungi, yeasts, and bacteria contribute to the oxidation, degradation, and conversion of tea components, resulting in characteristic changes in color, aroma, taste, and bioactive composition [13]. This microbial transformation promotes the formation of key components such as theabrownins, puerins, teadenol A, gallic acid, lovastatin, gamma-aminobutyric acid, and flavonoids like quercetin, which may contribute to the sensory quality and functional potential of Pu-erh tea [13].

Recent studies have reported that fermented teas like Pu-erh can positively impact metabolic health [14,15]. Abnormal glucose and lipid metabolism are key contributors to chronic diseases such as diabetes, obesity, and cardiovascular conditions, which are pressing global health concerns [16]. Tea polyphenols and flavonoids have been found to inhibit enzymes like α-amylase, α-glucosidase, and lipase, reducing the absorption of carbohydrates and lipids [17,18]. Moreover, tea components can bind bile acids and cholesterol, contributing to their excretion and overall hypolipidemic effects [19]. Notably, fermentation can amplify these biological activities by converting complex phenolic compounds into simpler, more bioavailable forms [20].

Building on these findings, applying the Pu-erh tea pile fermentation process to coffee leaves offers a promising approach for creating a novel coffee dark tea that integrates appealing sensory qualities with enhanced functional attributes. This method promotes the high-value utilization of coffee leaves while providing an environmentally sustainable and economically viable avenue for diversifying coffee-derived products. Accordingly, this study aimed to investigate the feasibility of producing dark tea from sun-dried coffee leaves (*Coffea arabica* L.) through the pile fermentation process characteristic of Pu-erh tea. The research focused on examining changes in physicochemical properties, tea pigments, and phytochemical profiles during fermentation; evaluating antioxidant activity; and assessing in vitro hypoglycemic and hypolipidemic effects, including enzyme inhibition, glucose diffusion, lipid-binding capacities, and sensory properties. The outcomes are expected to establish a theoretical and experimental foundation for the functional assessment and industrial application of fermented coffee leaf dark tea, fostering the sustainable use of coffee resources and the innovation of health-oriented beverages.

## 2. Materials and Methods

### 2.1. Culture Starters

*Saccharomyces cerevisiae* (ATCC 9763) and *Aspergillus niger* (CMCC 98003) were obtained from the Shanghai Bioresource Collection Center (Shanghai, China). *S. cerevisiae* was cultured in yeast extract peptone dextrose (YPD) broth at 28 °C for 3 days with shaking, harvested by centrifugation, washed with sterile saline (0.9%), and adjusted to 1 × 10^7^ cells/mL. *A. niger* was grown on potato dextrose agar (PDA) slants at 28 °C for 3 days, and spores were collected with 0.9% sterile saline, filtered through sterile cotton, and adjusted to 1 × 10^7^ cells/mL.

### 2.2. Pile Fermentation and Sample Preparation

**Figure 1:**
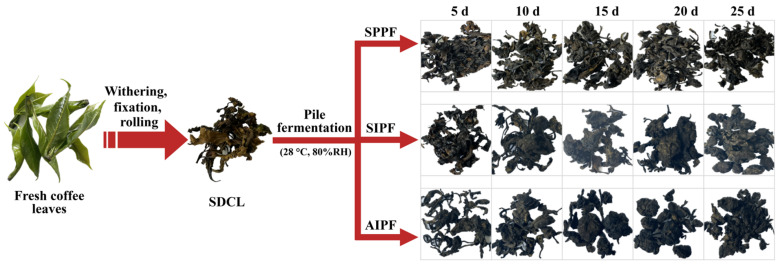
Sampling diagram. SDCL, sun-dried coffee leaves; SPPF, spontaneous pile fermentation; SIPF, *S. cerevisiae*-inoculated pile fermentation; AIPF, *A. niger*-inoculated pile fermentation.

The production process of the samples was in accordance with the standards of “Product of geographical indication—Puer tea” (GB/T 22111–2008) [21] (Figure 1). The raw material, sun-dried coffee leaves (SDCL), was supplied and processed by Baoshan Lushanyunshu Agricultural Development Co., Ltd. (Baoshan, China). The fresh leaves (Catimor CIFC 7963, *C. arabica* L.) were first spread out for withering for approximately 8 h, followed by fixation, rolling, and sun-drying to produce the SDCL, with a moisture content of about 8%. The SDCL underwent three types of pile fermentation: spontaneous pile fermentation (SPPF), *S. cerevisiae*-inoculated pile fermentation (SIPF), and *A. niger*-inoculated pile fermentation (AIPF). For the inoculated groups, two fungal spore suspensions (10^7^ spores/mL) were inoculated into the SDCL at a rate of 1.0%. For spontaneous fermentation, 0.9% sterile saline was used instead of inoculum as a control. Samples were adjusted to 35% moisture and fermented in a controlled chamber (28 °C, 80% relative humidity (RH)), with turning every 5 days. Subsamples were collected at 0, 5, 10, 15, 20, and 25 days from five locations within each pile and pooled. After air-drying, samples were split into three fractions: (1) intact leaves for sensory evaluation; (2) powders for physicochemical analysis; and (3) aqueous extracts for biochemical assays, prepared at 1:60 (*w*/*v*) with boiling water, followed by ultrasonic extraction and filtration (0.45 μm). All fractions were stored at −20 °C until analysis.

### 2.3. Determination of Basic Physicochemical Components

Dry matter (DM, %) was determined by oven-drying to constant weight at 103 °C (Tea—Preparation of ground sample and determination of dry matter content, GB/T 8303-2013) [22]. Water extract (WE, %) was measured by extracting 2 g tea with 300 mL boiling distilled water for 45 min, followed by drying the filtrate at 103 °C for 2 h (Tea—Determination of water extracts content, GB/T 8305-2013) [23]. Free amino acids (FAA, %) were determined through the ninhydrin colorimetric method (Tea—Determination of free amino acids contenta, GB/T 8314-2013) [24] using glutamic acid as the standard [25]. Briefly, 2 mL of sample or standard solution was mixed with 0.5 mL phosphate buffer (pH 8.0) and 0.5 mL 2% ninhydrin solution, heated in a boiling water bath for 15 min, diluted to 10 mL after cooling, and measured at 570 nm. Soluble sugars (SS, %) were determined through the phenol-sulfuric acid colorimetric method using glucose as the standard [25]. Briefly, 1 mL of sample or glucose standard solution was mixed with 1 mL of 50 g/L phenol solution and 5 mL of concentrated sulfuric acid, heated in a boiling water bath for 20 min, cooled to room temperature, and measured at 490 nm.

Tea pigments, including theabrownin (TB), theaflavin (TF), and thearubigin (TR), were analyzed following Wang et al. [26]. Dried powder (3.0 g) was extracted with 125 mL boiling water for 10 min, filtered, and cooled. The filtrate (50 mL) was partitioned with 50 mL ethyl acetate for 5 min. An aliquot of the ethyl acetate layer (4 mL) was diluted to 25 mL with 95% ethanol (solution A). The remaining ethyl acetate phase was shaken with 25 mL 2.5% NaHCO_3_ for 30 s, and 4 mL of the ethyl acetate layer was diluted to 25 mL with 95% ethanol (solution C). The aqueous layer (2 mL) was mixed with 2 mL saturated oxalic acid, 6 mL water, and 15 mL 95% ethanol (solution D). The second aqueous layer was treated similarly to obtain solution B. The absorbance of solutions A–D was read at 380 nm, and TB, TF, and TR were calculated using *E_A_*, *E_B_*, *E_C_*, and *E_D_* as follows:

(1) $$\mathrm{TBs}\;=\;\frac{2E_{B}\;\times \;7.06}{\mathrm{DM}}\;\times \;100\%$$

(2) $$\mathrm{TFs}=\frac{E_{C\;}\times \;2.25}{\mathrm{DM}}\;\times \;100\%$$

(3) $$\mathrm{TRs}=\frac{7.06\;\times \;(2E_{A}+2E_{D}\;-\;E_{C}\;-\;2E_{B})}{\mathrm{DM}}\;\times \;100\%$$

where *E_A_*, *E_B_*, *E_C_*, and *E_D_* represent absorbance values for solutions A–D, and DM is dry matter (%).

### 2.4. Quantitative Analysis of Phytochemical Compounds by HPLC

Phytochemicals in coffee dark tea extracts were quantified using high-performance liquid chromatography (HPLC) as described by Chen et al. [9] with modifications, including caffeine, trigonelline, mangiferin, rutin, and chlorogenic acids (5-caffeoylquinic acid (5-CQA), 3-caffeoylquinic acid (3-CQA), 4-caffeoylquinic acid (4-CQA), 3,5-dicaffeoylquinic acid (3,5-diCQA), and 3,4-dicaffeoylquinic acid (3,4-diCQA)). Separation was performed on a Phenomenex Kinetex C18 column (150 mm × 4.6 mm, 5 µm) using mobile phase A (H_2_O acidified with 0.1% (*v*/*v*) trifluoroacetic acid) and B (acetonitrile) with the following gradient: 0–10 min, 95–80% A; 10–12 min, 80% A; 12–15 min, 80–5% A; 15.1–16.5 min, 5% A; 16.6–19 min, 95% A. The column temperature was 25 °C, the flow rate was 1.5 mL/min, and the injection volume was 5 µL. Detection was conducted with a Waters 2998 Photodiode Array Detector at 264 nm (trigonelline), 280 nm (caffeine), 257 nm (mangiferin and rutin), and 325 nm (chlorogenic acids). External standard calibration was used for quantitative analysis, and the calibration curves showed good linearity within the tested concentration ranges (R^2^ > 0.99).

### 2.5. Determination of Total Phenolics and Flavonoids

Total phenolic content (TPC) was measured using the Folin–Ciocalteu assay with modifications [27]. Samples were diluted 25-fold, and 1 mL of diluted extract was mixed with 5 mL Folin–Ciocalteu reagent for 5 min, followed by addition of 4 mL 7.5% Na_2_CO_3_ and incubation for 60 min at room temperature. Absorbance was recorded at 765 nm, and TPC was calculated from a gallic acid standard curve and expressed as mg gallic acid equivalents (GAE)/g.

Total flavonoid content (TFC) was determined using the aluminum chloride method with modifications [28]. After 25-fold dilution, 1 mL of the sample was mixed with 0.5 mL of 10% aluminum nitrate for 5 min, followed by 2 mL of 4% NaCl and dilution to 10 mL with distilled water. After 15 min, absorbance was measured at 510 nm, and TFC was quantified using a rutin calibration curve, expressed as mg rutin equivalents (RE)/g.

### 2.6. Antioxidant Activity Assays

Antioxidant activity was evaluated using 2,2-diphenyl-1-picrylhydrazyl (DPPH) and 2,2′-azino-bis(3-ethylbenzothiazoline-6-sulfonic acid) (ABTS) assays with modifications [29,30]. For DPPH, 0.5 mL of sample was mixed with 4.5 mL DPPH solution and incubated for 30 min, and absorbance was measured at 519 nm. For ABTS, 1 mL of sample was mixed with 4 mL ABTS solution and incubated for 10 min, and absorbance was measured at 734 nm. The scavenging rate (%) was calculated as

(4) $$\mathrm{Scavenging}\;\mathrm{rate}\;(\%)=[1\;-\;\frac{{(A}_{s}\;-\;A_{b})}{A_{c}}]\;\times \;100\%$$

where A_s_, A_c_, and A_b_ are the absorbances of the sample, control, and blank, respectively. IC_50_ was determined from the regression curve of scavenging rate versus concentration. Trolox was used as a positive control.

### 2.7. Hypoglycemic Activity Assays

#### 2.7.1. α-Amylase and α-Glucoside Inhibition Activity

α-Amylase and *α*-glucosidase inhibition was measured using the methods of Ayyash et al. [31] with modifications. For *α*-amylase, 500 μL of sample was mixed with 500 μL *α*-amylase solution (0.02 mmol/mL, pH 6.9) and incubated at 37 °C for 10 min, followed by addition of 1% starch solution and incubation for 30 min. The reaction was terminated by adding 500 μL 3,5-dinitrosalicylic acid (DNS) reagent and boiling for 5 min. Absorbance was measured at 540 nm. For *α*-glucosidase, 50 μL of sample was mixed with 50 μL α-glucosidase (1 U/mL, pH 7.0) and incubated at 37 °C for 20 min, followed by 50 μL *p*-nitrophenyl-*α*-D-glucopyranoside (10 g/L) and incubation for another 30 min. The reaction was stopped by adding 100 μL Na_2_CO_3_, and absorbance was measured at 405 nm. Inhibition (%) was calculated as

(5) $$\mathrm{Inhibition}\;(\%)=[1\;-\;\frac{{(A}_{s}\;-\;A_{b})}{A_{c}}]\;\times \;100\%$$

where A_s_, A_c_, and A_b_ represent the absorbance of the sample, control, and blank, respectively. IC_50_ was determined from the regression curve of inhibition rate versus sample concentration.

#### 2.7.2. In Vitro Glucose Dialysis Retardation Capacity and Starch Digestibility

Glucose diffusion inhibition was measured following Ou et al. [32] with minor modifications. Briefly, 2 mL of sample was added to 10 mL of 100 mmol/L glucose solution and placed in a dialysis bag (molecular weight cut-off: 7000 Da). The bag was immersed in 200 mL deionized water and incubated at 37 °C with continuous shaking for 2.5 h. Dialysate (2 mL) was collected at 30 min intervals, and glucose concentration was measured using the DNS method. A control was run under the same conditions without sample.

Starch digestion inhibition was assessed using Benitez et al. [33] with slight modifications. α-Amylase solution (400 μL, 1 mg/mL) was added to 10 mL of 4% starch solution, followed by 2 mL of sample. The mixture was placed in a dialysis bag (molecular weight cut-off: 7000 Da) and incubated in 200 mL deionized water at 37 °C with continuous shaking for 2.5 h. Dialysate (2 mL) was collected every 30 min, and glucose concentration was determined using the DNS method. A control was conducted under the same conditions without sample.

### 2.8. Hypolipidemic Activity Assays

#### 2.8.1. Evaluation of Pancreatic Lipase Inhibitory Activity

Pancreatic lipase inhibition was assessed following Benitez et al. [33] with minor modifications. Briefly, 1 mL sample, 2 mL olive oil, 10 mL 0.1 mol/L phosphate buffer (pH 7.2), and 2 mL pancreatic lipase solution (0.75 mg/L) were mixed and incubated at 37 °C for 1.5 h. The reaction was stopped by placing the tubes in boiling water, and free fatty acids released were quantified through titration with 0.05 mol/L NaOH. Lipase inhibition (%) was calculated as the percentage reduction in free fatty acid production compared to the control.

#### 2.8.2. Sodium Cholate Binding Capacity

Sodium cholate binding capacity was assessed following Daou and Zhang [34] with minor modifications. Briefly, 2 mL sample was mixed with 4 mL 0.1 mol/L phosphate buffer (pH 6.8) and 4 mL 0.5 mmol/L sodium cholate solution, and incubated at 37 °C with shaking for 2 h. The mixture was centrifuged at 800× *g* for 20 min, and 2.5 mL supernatant was mixed with 7.5 mL 60% sulfuric acid, incubated at 70 °C for 20 min, and cooled. Absorbance was measured at 620 nm. Binding capacity was calculated based on the concentration difference before and after the reaction, using sodium cholate as the standard.

#### 2.8.3. Cholesterol Binding Capacity

Cholesterol binding capacity was assessed following Daou and Zhang [34] with minor modifications. Fresh egg yolk was diluted (1:9, *w*/*w*) with distilled water and homogenized to form an emulsion. Then, 0.5 mL of sample was mixed with 10 mL egg yolk emulsion, and the pH was adjusted to 2.0 and 7.0, respectively. The mixtures were incubated at 37 °C for 2 h with shaking and centrifuged at 800× *g* for 15 min. The supernatant was diluted fivefold with 90% acetic acid and reacted with o-phthalaldehyde for color development at 25 °C for 10 min. Absorbance was measured at 550 nm, and cholesterol binding capacity was calculated from a standard curve.

### 2.9. Sensory Evaluation

Sensory evaluation of coffee leaf dark tea followed the “Methodology for sensory evaluation of tea” (GB/T 23776–2018) [35]. All participants provided written informed consent before the study. A panel of ten trained tasters (five men and five women) assessed the samples based on five attributes: appearance (20%), liquor color (15%), aroma (25%), taste (30%), and infused leaves (10%). Before formal evaluation, the panelists were trained and familiarized with the sensory attributes, scoring criteria, and terminology. Samples were coded with random three-digit numbers and presented in a randomized and blinded order. For each test, 3.0 g of tea was brewed with 150 mL boiling water for 2 min, then poured into cups for evaluation. A second infusion was brewed for 5 min. The comprehensive sensory score was calculated using the specified weights. Appearance, liquor color, and infused leaves were documented through photographs, while aroma and taste were recorded descriptively. Sensory terminology followed GB/T 14487–2017 [36]. Inter-panelist consistency was checked before data analysis to ensure the reliability of the sensory results.

### 2.10. Statistical Analysis

All experiments were conducted in triplicate, and results are expressed as mean ± SD. Statistical analysis was performed using one-way analysis of variance (ANOVA) followed by Tukey’s multiple comparison test (*p* < 0.05) with SPSS 26.0 (IBM Corp., Armonk, NY, USA). Data visualization was done using Origin 9.65 (OriginLab Corp., Northampton, MA, USA).

## 3. Result and Discussion

### 3.1. Basic Chemical Compositions of Coffee Leaf Dark Tea

Tea water extracts are the soluble substances in tea, such as polyphenols, alkaloids, terpenoids, flavonoids, amino acids, and vitamins, which contribute primarily to the flavor of tea infusions [11]. This study examined the changes in the water extract content of coffee dark tea during different stages of pile fermentation (Table 1). The results show a decrease in water extract content as fermentation progresses. After 25 days of fermentation, the water extract content of coffee leaf dark tea significantly decreased (*p* < 0.05). Compared to the unfermented sample (SDCL, 588.42 mg/g), the values for the fermented treatments were 446.13 mg/g for SPPF, 399.35 mg/g for SIPF, and 442.50 mg/g for AIPF. This change suggests that differences in microbial metabolism during fermentation lead to variations in the water-soluble chemical components, significantly affecting the chemical profile of the tea. Thus, the type of microorganisms and their metabolic processes are key factors influencing the changes in water extract components.

Soluble sugars, as a key sweet substance in tea infusions, play a significant role in shaping its taste characteristics [37]. As shown in Table 1, the soluble sugar content of coffee leaf dark tea processed through SPPF decreases from 36.54 mg/g (SDCL) to 29.56 mg/g. In contrast, coffee leaf dark tea processed with SIPF and AIPF shows an increasing trend in soluble sugar content, rising to 46.64 and 71.73 mg/g, respectively. These results highlight that external microorganisms during fermentation influence soluble sugar levels. Fungal enzymes, such as cellulase and pectinase, contribute to this increase, although the sugars are also utilized by microorganisms as a carbon source [38]. Notably, AIPF shows consistently higher soluble sugar content than SIPF at the same fermentation stage, which may be associated with differences in microbial metabolism and enzyme secretion; however, this explanation remains speculative without direct microbial or enzymatic evidence.

Amino acids are crucial for the taste and aroma of tea, contributing to bitterness, astringency, sourness, sweetness, and umami [39]. The free amino acid content of coffee leaf dark tea shows a decreasing trend during pile fermentation. By the end of the 25-day fermentation period, the free amino acid content of teas processed via SPPF, SIPF, and AIPF decreased from 31.81 mg/g (SDCL) to 11.57, 13.02, and 13.74 mg/g, respectively. The entire pile fermentation process significantly reduced the free amino acid content (*p* < 0.05), a result consistent with findings from Pu-erh tea fermentation, where free amino acids are consumed as a nitrogen source [40]. Compared with SPPF, the inoculation of exogenous fungi (SIPF and AIPF) significantly slowed the rate of amino acid depletion. However, the differences in the degree of reduction between the two inoculated treatments suggest that their distinct growth and propagation patterns may influence the rate at which amino acids are consumed during fermentation.

### 3.2. Tea Pigments of Coffee Leaf Dark Tea

Tea color and mouthfeel are strongly influenced by major pigments, including thearubigins (TRs), theaflavins (TFs), and theabrownins (TBs) [41]. As shown in Table 1, the content of TRs in coffee leaf dark tea decreases during pile fermentation. Specifically, the TR content in coffee leaf dark tea processed with SPPF, SIPF, and AIPF treatments decreased from 22.19 mg/g in SDCL to 14.28, 10.64, and 9.14 mg/g, respectively. Similarly, the content of TFs in the fermented coffee leaf dark tea also showed a downward trend, with TF content decreasing from 1.00 mg/g in SDCL to 0.47, 0.50, and 0.50 mg/g for the three treatments. In contrast, TBs increased after pile fermentation, rising from 73.44 mg/g in SDCL to 89.90, 112.50, and 120.05 mg/g for the SPPF, SIPF, and AIPF treatments, respectively. Among these pigments, TBs, a hallmark bioactive fraction in Pu-erh tea, are typically generated through microbial fermentation and/or enzymatic oxidation during dark tea processing [42]. Recent studies further indicate that TBs are associated with multiple health-related activities, including anti-obesity, anti-diabetic, anti-inflammatory, antioxidant, anti-cancer, anti-photodamage, and gut microbiota modulation effects [43]. Overall, these trends suggest that pile fermentation can enrich TBs and may strengthen the functional potential of coffee leaf dark tea.

### 3.3. Phytochemical Profile of Coffee Leaf Dark Tea

Caffeine and trigonelline are two major alkaloids found in coffee leaves, with higher concentrations in young leaves compared to mature ones [9]. During pile fermentation, both alkaloids exhibit distinct patterns across different treatments (Table 2). For caffeine, SPPF shows a steady decline from 18.34 mg/g at day 0 to 14.63 mg/g by day 25, while SIPF maintains a relatively stable level, decreasing slightly from 18.67 mg/g to 16.40 mg/g over the same period. AIPF starts with the highest caffeine content (20.79 mg/g at day 5) and decreases gradually to 14.94 mg/g by day 25. In contrast, trigonelline shows a sharper decrease under SPPF, dropping from 9.92 mg/g at day 0 to 1.46 mg/g by day 25. SIPF results in a slower decline, with trigonelline content decreasing from 10.14 mg/g to 6.02 mg/g by day 25, while AIPF starts with the highest content (11.87 mg/g at day 5) and decreases to 3.13 mg/g by day 25. In summary, both caffeine and trigonelline decrease during fermentation, with SPPF causing the most significant reductions. SIPF and AIPF lead to slower declines, with AIPF maintaining the highest levels of both compounds throughout fermentation. Trigonelline, in particular, has gained attention as a potential health-promoting dietary component [44]. The use of exogenous microbial inoculation in fermentation helps preserve trigonelline and reduce its degradation.

Mangiferin, rutin, and caffeoylquinic acids (CQAs) are major bioactive constituents of coffee leaves [45]. Their levels and fermentation-induced changes in coffee leaf dark tea are summarized in Table 2. Mangiferin decreases gradually under all treatments, with SPPF causing the most significant reduction, followed by SIPF and AIPF, which exhibit slower declines. Similarly, rutin shows a steady decrease across all treatments, with SPPF resulting in the largest reduction, while SIPF and AIPF lead to more gradual decreases. The CQAs experience substantial reductions, especially under SPPF, where the levels of 5-CQA, 3-CQA, and 4-CQA drop to near zero by day 25. SIPF and AIPF also lead to declines, but at a slower rate, with AIPF maintaining higher levels of these compounds throughout fermentation. In conclusion, all these compounds show a decrease during fermentation, with SPPF causing the most dramatic reductions, while SIPF and AIPF result in slower declines, indicating that different fermentation treatments influence the degradation and preservation of bioactive compounds in coffee leaf dark tea.

### 3.4. TPC, TFC and Antioxidant Activity of Coffee Leaf Dark Tea

During Pu-erh pile fermentation, TPC and TFC decrease significantly with increasing fermentation time [46], and this trend is also observed in the processing of coffee leaf dark tea processed through SPPF (Table 3). In contrast, coffee leaf dark tea fermented using SIPF and AIPF shows a different trend: TPC and TFC initially increase, followed by a decrease in the later stages of fermentation. Specifically, the SIPF coffee leaf dark tea reaches its highest levels of TPC (23.60 mg GAE/g) and TFC (10.65 mg RE/g) after 10 days of fermentation. Meanwhile, the AIPF coffee leaf dark tea peaks at 5 days, with a TPC of 22.44 mg GAE/g and TFC of 10.13 mg RE/g. In Pu-erh fermentation, extracellular enzymes produced by yeast and Aspergillus can promote transformations of phenolic-related substrates and favor the accumulation of certain phenolic acids [13]. This phenomenon may be associated with microbial or enzymatic transformations during the early stages of SIPF and AIPF. However, because microbial population dynamics and extracellular enzyme activities were not measured, the underlying mechanisms require further investigation. Such shifts in phenolic metabolism reflect the distinct microbial and enzymatic environments of different fermentation modes and may influence product sensory attributes. As tea polyphenols strongly affect bitterness and astringency [47], changes in TPC and TFC during fermentation are likely critical in shaping the flavor and overall quality of coffee leaf dark tea.

Antioxidant capacity was evaluated using DPPH and ABTS assays, which probe complementary radical-scavenging behaviors [48]. Half-maximal inhibitory concentration (IC_50_) values for DPPH and ABTS radical-scavenging by coffee leaf dark tea were determined, with lower IC_50_ values indicating stronger radical-scavenging activity (Table 3). The antioxidant activity of SPPF samples exhibited a trend consistent with the changes in TPC and TFC. For SIPF and AIPF, antioxidant activity reached its maximum on days 10 and 5, respectively, with IC_50_ values of 33.68 mg/mL and 34.20 mg/mL for DPPH, and 65.75 mg/mL and 67.79 mg/mL for ABTS. Overall, the co-variation in TPC/TFC and IC_50_ supports a close linkage between phenolic dynamics and antioxidant performance, highlighting fermentation mode as a key driver of chemical and functional properties in coffee leaf dark tea.

### 3.5. Hypoglycemic Activities of Coffee Leaf Dark Tea

α-Amylase and α-glucosidase are key enzymes in carbohydrate digestion, and inhibiting them helps delay intestinal carbohydrate absorption [49]. As shown in Table 3, the α-glucosidase inhibitory activities of coffee leaf dark tea extracts were evaluated, with lower IC_50_ values indicating stronger inhibition. The α-amylase inhibitory activity of SPPF extracts gradually decreased during fermentation, whereas SIPF and AIPF extracts exhibited an initial increase followed by a decline. SIPF samples reached their highest inhibitory activity on day 10 (IC_50_ = 1.48 mg/mL), while AIPF samples peaked on day 5 (IC_50_ = 1.61 mg/mL). A similar pattern was observed for α-glucosidase inhibition: SIPF samples showed maximum activity on day 10 (IC_50_ = 1.15 mg/mL), and AIPF samples peaked on day 5 (IC_50_ = 1.53 mg/mL). These results indicate that, given appropriate microbial inoculation, optimizing fermentation duration can effectively enhance the in vitro α-amylase and α-glucosidase inhibitory activities of coffee leaf dark tea. Considering that the unique microbial communities involved in Pu-erh tea piling fermentation are known to produce bioactive compounds (such as peptide-based inhibitors) that contribute to α-amylase and α-glucosidase inhibition [50], similar microbially driven mechanisms may also operate during the fermentation of coffee leaf dark tea.

**Figure 2:**
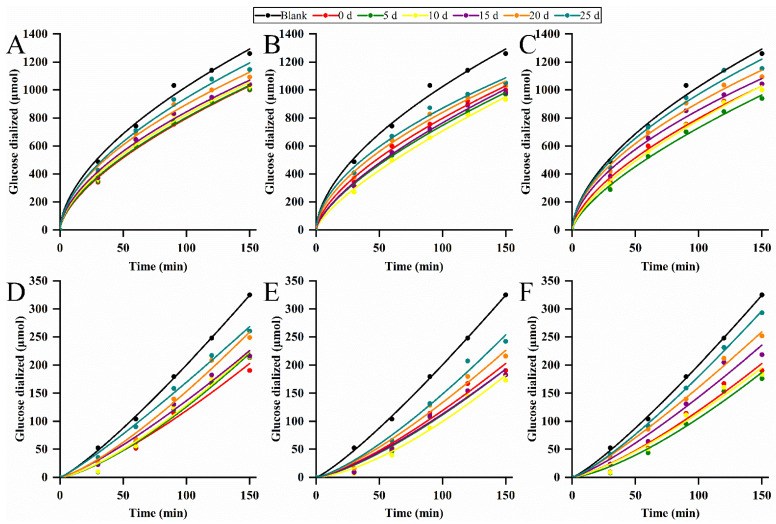
Effect of coffee leaf dark tea on glucose diffusion ((**A**): SPPF; (**B**): SIPF; (**C**): AIPF) and starch digestibility ((**D**): SPPF; (**E**): SIPF; (**F**): AIPF) during 0 to 150 min of incubation at 37 °C. SPPF, spontaneous pile fermentation; SIPF, *S. cerevisiae*-inoculated pile fermentation; AIPF, *A. niger*-inoculated pile fermentation.

The glucose dialysis retardation index (GDRI) provides an in vitro proxy for limiting glucose diffusion/absorption [51]. As shown in Figure 2A–C, glucose concentrations in the dialysates increased over time (30–120 min) for all treatments; however, the presence of coffee leaf dark tea extracts significantly reduced glucose diffusion compared with the blank control (*p* < 0.05). Among the fermented products, SIPF showed a greater retardation of glucose diffusion than AIPF, possibly due to the higher soluble sugar content in AIPF, which accelerates diffusion rather than adsorbing glucose. Within the SPPF, SIPF, and AIPF groups, samples corresponding to 0 d, 10 d, and 5 d of fermentation, respectively, exhibited the strongest inhibitory effects on glucose diffusion. These findings suggest that appropriately fermented coffee leaf dark tea may possess desirable glucose-modulating properties, which could support its potential application in the development of hypoglycemic functional products.

Starch digestibility is a key metabolic factor influencing postprandial blood glucose levels [52]. As shown in Figure 2D–F, the coffee leaf dark tea extracts obtained from different fermentation processes significantly reduced starch digestibility in vitro. After 150 min of dialysis, the glucose concentrations in all extract-treated systems were markedly lower than in the blank control, likely due to their higher α-amylase inhibitory activity, which limits starch hydrolysis and consequently slows glucose diffusion. The maximum starch digestion inhibition rates were 41.38% for SPPF, 46.77% for SIPF, and 45.85% for AIPF. Within each processing method, the strongest inhibition occurred in the 0-day (SPPF), 10-day (SIPF), and 5-day (AIPF) samples, respectively. Previous studies have shown that polyphenols can reduce starch digestibility by inhibiting amylase activity [53,54], and the present results are consistent with this mechanism, as the inhibition rates corresponded closely with polyphenol levels. Overall, these data indicate that coffee leaf dark tea could serve as a natural ingredient for moderating postprandial glucose.

### 3.6. Hypolipidemic Activity of Coffee Leaf Dark Tea

**Figure 3:**
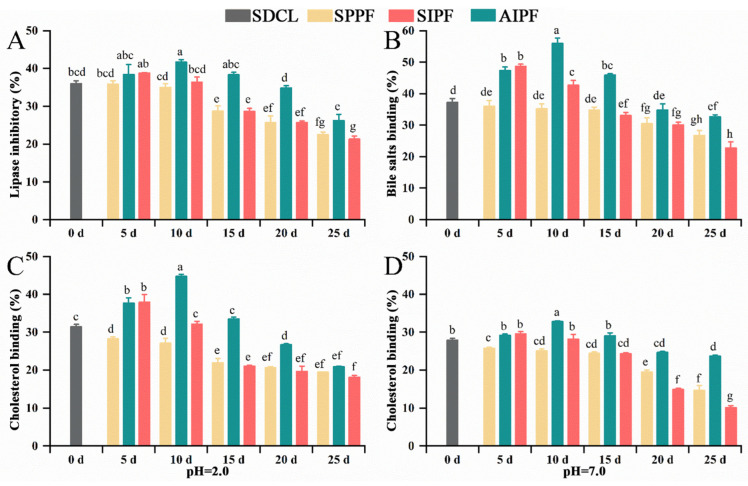
Effect of coffee leaf dark tea on lipase inhibitory activity (%) (**A**), bile salt binding capacity (%) after 2 h (**B**), and cholesterol binding capacity (%) at pH 2.0 (**C**) and pH 7.0 (**D**). SPPF, spontaneous pile fermentation; SIPF, *S. cerevisiae*-inoculated pile fermentation; AIPF, *A. niger*-inoculated pile fermentation. Different letters in the same row indicate a statistically significant difference (*p* < 0.05).

As shown in Figure 3A, all coffee leaf dark tea extracts inhibited pancreatic lipase activity, with SIPF samples exhibiting the strongest inhibition and reaching a maximum of 41.71% on day 10. During fermentation, lipase inhibition decreased in SPPF samples, whereas SIPF and AIPF samples showed an initial increase followed by a decline, with the turning point occurring earlier in AIPF. Since tea-related hypolipidemic activity has been linked to phenolic composition and the affinity of phenolic acids for pancreatic lipase [55], the observed inhibition in coffee leaf dark tea is plausibly associated with its polyphenol pool and their interactions with lipase (Figure 3A, Table 3). Together, these findings support the potential of coffee leaf dark tea as a functional ingredient for lipid regulation.

Bile salt binding is considered a mechanism for cholesterol reduction because enhanced fecal excretion promotes continual conversion of cholesterol into bile acids and may lower circulating cholesterol [56,57]. Therefore, substances with bile salt binding capacity can be identified as having lipid-lowering activity. The changes in bile salt binding capacity of coffee leaf dark tea extract during the pile fermentation process are shown in Figure 3B. The coffee leaf dark tea extract from SPPF exhibited a decreasing trend in bile salt binding capacity, while the extracts from SIPF and AIPF showed an initial increase followed by a decline. Throughout the fermentation cycle, the coffee leaf tea from SIPF reached its maximum bile salt-binding capacity of 55.97% on day 10 of fermentation, whereas the coffee leaf tea from AIPF reached its peak at 48.67% on day 5. Polyphenols (such as catechins and epicatechins) and tea pigments (such as theaflavins and thearubigins) have been reported to bind bile salts [56], and the positive association observed here between bile salt binding and TPC suggests that phenolic-related components contribute to this effect, reinforcing the cholesterol-lowering potential of coffee leaf dark tea.

Cholesterol binding capacity of coffee leaf dark tea extracts from different pile fermentation methods was evaluated under pH 7.0 and 2.0 conditions to simulate the intestinal and gastric environments. The results demonstrated that pH significantly influenced the cholesterol adsorption capacity of the extract. Specifically, under acidic conditions, the coffee leaf dark tea extract exhibited a higher cholesterol adsorption capacity, reaching a maximum of 44.76%. Given that the pH of the human stomach is approximately 2 and that of the small intestine is around 7, these findings suggest that the cholesterol binding capacity of coffee leaf dark tea is greater in the stomach than in the small intestine. This effect may be attributed to the types of polyphenols present in the tea. Under acidic conditions, polyphenols are predominantly present in the form of phenolic hydroxyl and hydroxyl groups, which enhance adsorption. In contrast, under neutral conditions, phenolic hydroxyl groups exist in salt form, leading to a significant reduction in their ability to bind cholesterol.

However, these findings are limited to in vitro assays and should not be directly interpreted as evidence of in vivo hypoglycemic or hypolipidemic efficacy. Further studies involving bioavailability evaluation, animal experiments, and clinical validation are necessary.

### 3.7. Sensory Evaluation of Coffee Leaf Dark Tea

**Figure 4:**
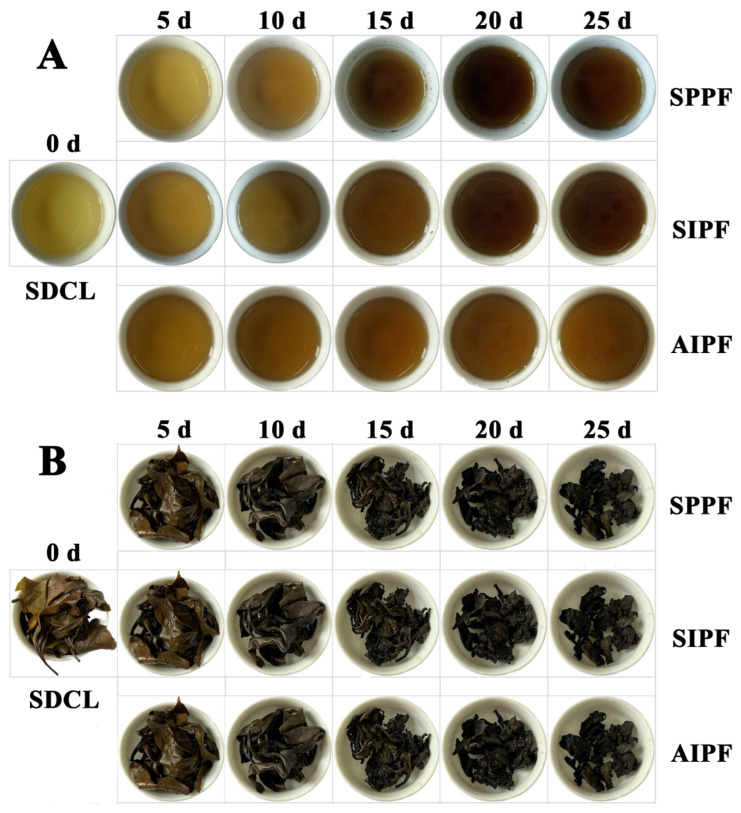
Liquor color (**A**) and infused leaves (**B**) of coffee leaf dark tea. SDCLs, sun-dried coffee leaves; SPPF, spontaneous pile fermentation; SIPF, *S. cerevisiae*-inoculated pile fermentation; AIPF, *A. niger*-inoculated pile fermentation.

Coffee leaf dark tea samples were subjected to sensory evaluation, as shown in Figure 1 and Figure 4 and Table 4. In terms of appearance, prolonging the pile fermentation time led to increasingly compact leaves, and inoculation with yeast or *A. niger* further accelerated the fermentation process, resulting in an even tighter leaf appearance and develop “golden flowers” (*Eurotium cristatum*). The SIPF sample fermented for 10 days obtained the highest appearance score (88.44). In terms of tea liquor color, the glossiness of the SIPF and AIPF samples was higher than that of the SPPF, which is consistent with the experimental results on theaflavins. The AIPF sample fermented for 15 days received the highest score of 90.33, and its tea liquor consistently outperformed both SIPF and SPPF samples in color evaluation. In terms of aroma, the AIPF samples for 10 days achieved the highest aroma score of 85.00. The SIPF samples exhibited a pronounced “Qu” fermented aroma, whereas the AIPF samples were characterized by fruity and woody notes, indicating that inoculation with exogenous strains during pile fermentation imparts strain-specific aroma characteristics to coffee leaf dark tea. In terms of taste, the AIPF samples for 15 days achieved the highest taste score of 86.00. Compared with the unfermented sample (SDCL), pile fermentation promoted the transformation of bitter and astringent components in coffee leaves into other flavor substances, thereby reducing bitterness, and inoculation with exogenous strains further accelerated this process. In terms of infused leaves, the AIPF sample fermented for 10 days achieved the highest score of 89.00. These results suggest that pile fermentation makes the infused leaves of coffee leaf dark tea more compact, which may be associated with microbial activity during fermentation.

According to the total sensory scores, the unfermented sample (SDCL) showed the lowest overall quality (75.01). SPPF exhibited a slight improvement (mean score 76.58), whereas SIPF or AIPF led to markedly higher overall scores, with mean values of 80.57 and 83.23, respectively. Across all treatments, the highest sensory quality was generally observed between 10 and 15 days of fermentation, with the AIPF sample fermented for 15 days achieving the highest total score (86.24), SIPF at 10 days (84.15), and SPPF at 15 days (79.14). When fermentation was extended to 25 days, the total scores declined to varying degrees, indicating that appropriate pile fermentation duration combined with microbial inoculation is critical for optimizing the overall sensory quality of coffee leaf dark tea, whereas over-fermentation may compromise its sensory attributes. Overall, these findings demonstrate that appropriate pile fermentation time combined with targeted microbial inoculation substantially enhances the sensory quality of coffee leaf dark tea, whereas excessive fermentation tends to diminish its overall acceptability.

## 4. Conclusions

This study demonstrated that sun-dried Arabica coffee leaves can be successfully processed into coffee leaf dark tea using Pu-erh-style pile fermentation. Pile fermentation markedly altered the physicochemical composition, tea pigments and phytochemical profile of coffee leaves, while inoculated fermentations with *S. cerevisiae* (SIPF) and *A. niger* (AIPF) better preserved soluble sugars, alkaloids and key phenolic compounds, and promoted the formation of theabrownins compared with SPPF.

Fermentation mode and duration strongly influenced in vitro antioxidant capacity and hypoglycemic- and hypolipidemic-related activities, which generally increased and then declined, with SIPF and AIPF achieving superior bioactivity at appropriate intermediate fermentation times. Sensory evaluation further confirmed that inoculated fermentations, particularly AIPF and SIPF at 10–15 days, produced coffee leaf dark tea with higher overall quality, characterized by brighter liquor color, pleasant fruity–woody aroma, mellow taste with after-sweetness, and compact infused leaves. Overall, targeted microbial inoculation combined with controlled pile fermentation offers a promising approach to enhance both the functional properties and sensory quality of coffee leaf dark tea and support its potential as a high-value functional beverage. However, the present study did not include microbial population analysis, enzyme activity assays, or in vivo evaluation. Therefore, further studies are needed to clarify the fermentation mechanisms and confirm the physiological relevance of the observed activities.

## Figures and Tables

**Table 1:** Basic chemical compositions and tea pigments of coffee leaf dark tea (mg/g).

Sample	PF Days	Basic Chemical Compositions			Tea Pigments		
		WE	SS	FAA	TRs	TFs	TBs
SDCL	0	588.42 ± 22.20 a	36.54 ± 0.15 i	31.81 ± 0.43 a	22.19 ± 0.57 a	1.00 ± 0.01 a	73.44 ± 2.75 f
SPPF	5	584.95 ± 29.63 a	36.57 ± 0.81 i	17.25 ± 0.34 ef	22.15 ± 0.38 a	0.84 ± 0.03 c	75.74 ± 5.62 f
	10	554.67 ± 11.11 abc	35.40 ± 0.14 j	15.91 ± 0.45 h	15.54 ± 0.17 cd	0.60 ± 0.01 ef	77.25 ± 0.47 ef
	15	496.13 ± 4.14 cdef	34.22 ± 0.04 k	13.77 ± 0.35 j	15.25 ± 0.16 cd	0.59 ± 0.01 fg	77.84 ± 2.80 ef
	20	480.79 ± 26.85 defg	33.27 ± 0.15 k	13.04 ± 0.24 j	15.14 ± 0.44 cd	0.51 ± 0.00 i	86.85 ± 1.07 cd
	25	446.13 ± 22.61 efgh	29.56 ± 0.13 l	11.57 ± 0.25 k	14.28 ± 0.5 de	0.47 ± 0.01 l	89.90 ± 1.32 bcd
SIPF	5	557.06 ± 9.04 abc	37.49 ± 0.32 hi	18.01 ± 0.19 e	18.34 ± 0.36 b	0.99 ± 0.01 a	78.15 ± 2.67 ef
	10	508.62 ± 32.69 bcde	37.74 ± 0.93 h	17.81 ± 0.24 e	13.67 ± 0.62 ef	0.73 ± 0.01 d	87.74 ± 1.13 cd
	15	463.76 ± 24.88 defgh	39.81 ± 0.20 g	16.35 ± 0.12 gh	12.61 ± 0.27 f	0.61 ± 0.01 ef	91.86 ± 0.27 bc
	20	427.82 ± 22.73 gh	41.41 ± 0.08 f	14.71 ± 0.14 i	11.24 ± 0.25 gh	0.53 ± 0.01 gh	92.18 ± 1.02 bc
	25	399.35 ± 14.51 h	46.64 ± 0.04 e	13.02 ± 0.31 j	10.64 ± 0.60 h	0.50 ± 0.02 ij	112.50 ± 3.64 a
AIPF	5	567.89 ± 22.26 ab	47.12 ± 0.12 e	27.31 ± 0.34 c	16.11 ± 0.47 c	0.90 ± 0.01 b	84.01 ± 2.82 de
	10	527.38 ± 7.43 abcd	53.47 ± 0.43 d	28.87 ± 0.41 b	12.60 ± 0.34 f	0.62 ± 0.01 e	91.65 ± 2.31 bcd
	15	488.15 ± 33.61 defg	56.21 ± 0.31 c	19.03 ± 0.50 d	12.37 ± 0.84 fg	0.60 ± 0.00 f	96.41 ± 0.37 b
	20	476.63 ± 15.83 defg	67.97 ± 0.10 b	16.80 ± 0.26 fg	10.42 ± 0.24 h	0.52 ± 0.00 hi	116.16 ± 3.93 a
	25	442.50 ± 15.52 fgh	71.73 ± 0.24 a	13.74 ± 0.32 j	9.14 ± 0.51 i	0.50 ± 0.01 jk	120.05 ± 1.47 a

Note: Values are expressed as mean ± SD (n = 3). Mean values within a column followed by different letters are significantly different according to Tukey’s test (*p* < 0.05). PF, pile fermentation; SDCLs, sun-dried coffee leaves; SPPF, spontaneous pile fermentation; SIPF, *S. cerevisiae*-inoculated pile fermentation; AIPF, *A. niger*-inoculated pile fermentation; WE, water extract; FAA, free amino acid; SS, soluble sugar; TFs, theaflavins; TRs, thearubigins; TBs, theabrownins.

**Table 2:** Changes in phytochemicals in coffee leaf dark tea during pile fermentation (mg/g).

Sample	PF Days	Caffeine	Trigonelline	Mangiferin	Rutin	5-CQA	3-CQA	4-CQA	3,5-CQA	3,4-CQA
SDCL	0	18.34 ± 0.04 abc	9.92 ± 0.64 b	5.08 ± 0.72 abc	1.49 ± 0.08 bcd	9.91 ± 0.21 a	0.43 ± 0.01 b	1.20 ± 0.01 a	1.04 ± 0.03 b	0.98 ± 0.03 d
SPPF	5	18.15 ± 0.03 abcd	9.87 ± 0.09 b	4.87 ± 0.11 abc	1.33 ± 0.04 cde	9.68 ± 0.23 a	0.99 ± 0.01 a	1.97 ± 0.02 b	1.16 ± 0.02 a	2.32 ± 0.00 a
	10	16.37 ± 2.61 bcd	5.41 ± 0.27 cd	4.51 ± 0.7 bcd	1.33 ± 0.43 cde	1.05 ± 0.03 d	0.33 ± 0.00 bc	0.51 ± 0.01 c	0.45 ± 0.04 c	1.18 ± 0.02 c
	15	15.07 ± 2.39 bcd	3.62 ± 0.03 de	4.00 ± 0.77 cd	1.07 ± 0.25 def	0.10 ± 0.00 e	0.00 ± 0.00 d	0.16 ± 0.06 d	0.00 ± 0.00 e	0.00 ± 0.00 f
	20	16.13 ± 0.22 bcd	4.30 ± 0.40 de	3.20 ± 0.12 de	0.95 ± 0.05 ef	0.04 ± 0.00 e	0.00 ± 0.00 d	0.23 ± 0.01 e	0.00 ± 0.00 e	0.00 ± 0.00 f
	25	14.63 ± 1.18 d	1.46 ± 0.11 f	3.07 ± 0.08 de	0.90 ± 0.11 ef	0.00 ± 0.00 e	0.00 ± 0.00 d	0.25 ± 0.04 f	0.00 ± 0.00 e	0.00 ± 0.00 f
SIPF	5	18.67 ± 1.04 ab	10.14 ± 0.86 b	5.73 ± 0.60 ab	1.82 ± 0.30 abc	8.18 ± 0.50 b	1.24 ± 0.36 a	1.66 ± 0.07 f	0.97 ± 0.03 b	1.79 ± 0.02 b
	10	18.13 ± 0.63 abcd	9.59 ± 1.10 b	6.06 ± 0.21 a	2.14 ± 0.01 a	0.34 ± 0.02 e	0.08 ± 0.01 cd	0.18 ± 0.02 f	0.16 ± 0.02 d	0.47 ± 0.02 e
	15	18.08 ± 0.95 abcd	8.96 ± 0.16 b	5.17 ± 0.35 abc	1.75 ± 0.17 abc	0.18 ± 0.00 e	0.11 ± 0.01 cd	0.27 ± 0.00 fg	0.20 ± 0.01 d	0.47 ± 0.02 e
	20	17.52 ± 0.4 abcd	6.55 ± 0.63 c	4.23 ± 0.01 cd	1.31 ± 0.23 cde	0.07 ± 0.01 e	0.00 ± 0.00 d	0.16 ± 0.02 fg	0.00 ± 0.00 e	0.00 ± 0.00 f
	25	16.40 ± 1.44 bcd	6.02 ± 0.60 c	3.24 ± 0.04 de	1.04 ± 0.07 def	0.00 ± 0.00 e	0.00 ± 0.00 d	0.28 ± 0.03 fgh	0.00 ± 0.00 e	0.00 ± 0.00 f
AIPF	5	20.79 ± 0.32 a	11.87 ± 0.32 a	5.89 ± 0.04 ab	1.94 ± 0.01 ab	1.83 ± 0.15 c	0.53 ± 0.02 b	0.71 ± 0.02 fgh	0.51 ± 0.11 c	1.81 ± 0.19 b
	10	17.67 ± 1.47 abcd	8.55 ± 0.04 b	5.14 ± 0.73 abc	1.61 ± 0.02 bc	0.00 ± 0.00 e	0.05 ± 0.00 d	0.12 ± 0.03 ghi	0.18 ± 0.01 d	0.43 ± 0.03 e
	15	17.32 ± 0.29 abcd	6.45 ± 0.26 c	3.70 ± 0.45 cd	1.05 ± 0.12 def	0.00 ± 0.00 e	0.00 ± 0.00 d	0.33 ± 0.00 hi	0.00 ± 0.00 e	0.00 ± 0.00 f
	20	15.40 ± 1.40 bcd	4.07 ± 1.01 de	3.15 ± 0.95 de	0.90 ± 0.07 ef	0.00 ± 0.00 e	0.00 ± 0.00 d	0.27 ± 0.05 hi	0.00 ± 0.00 e	0.00 ± 0.00 f
	25	14.94 ± 0.45 cd	3.13 ± 0.02 de	2.05 ± 0.02 e	0.74 ± 0.01 f	0.00 ± 0.00 e	0.00 ± 0.00 d	0.29 ± 0.01 i	0.00 ± 0.00 e	0.00 ± 0.00 f

Note: Values are expressed as mean ± SD (n = 3). Mean values within a column followed by different letters are significantly different according to Tukey’s test (*p* < 0.05). PF, pile fermentation; SDCLs, sun-dried coffee leaves; SPPF, spontaneous pile fermentation; SIPF, *S. cerevisiae*-inoculated pile fermentation; AIPF, *A. niger*-inoculated pile fermentation; 3-CQA, 3-caffeoylquinic acid; 4-CQA, 4-caffeoylquinic acid; 5-CQA, 5-caffeoylquinic acid; 3,4-CQA, 3,4-dicaffeoylquinic acid; 3,5-CQA, 3,5-dicaffeoylquinic acid.

**Table 3:** Bioactive components, antioxidant activities (IC_50_) and hypoglycemic activities (IC_50_) of coffee leaf dark tea.

Sample	PF Days	Bioactive Components		Antioxidant Activities		Hypoglycemic Activities	
		TPC (mg GAE/g)	TFC (mg RE/g)	DPPH (mg/mL)	ABTS (mg/mL)	*α*-Amylase (mg/mL)	*α*-Glucosidase (mg/mL)
Trolox	-	-	-	2.64 ± 0.06 j	5.24 ± 0.10 m	-	-
Acarbose	-	-	-	-	-	0.54 ± 0.01 k	0.82 ± 0.01 k
SDCL	0	19.60 ± 1.87 bcde	8.87 ± 0.22 cde	37.60 ± 0.18 ef	70.77 ± 0.39 gh	1.82 ± 0.01 e	1.75 ± 0.03 f
SPPF	5	19.54 ± 0.12 bcde	8.85 ± 0.20 de	37.91 ± 0.31 e	71.02 ± 0.33 gh	1.83 ± 0.01 e	1.75 ± 0.00 f
	10	19.25 ± 0.91 bcdef	8.75 ± 0.06 de	38.19 ± 0.25 e	71.34 ± 0.19 g	1.88 ± 0.01 d	1.77 ± 0.01 ef
	15	17.46 ± 0.26 defg	7.95 ± 0.09 f	39.69 ± 0.05 cd	72.59 ± 0.31 f	1.89 ± 0.01 d	1.79 ± 0.01 de
	20	16.88 ± 1.40 defg	6.93 ± 0.14 h	41.01 ± 0.10 b	77.06 ± 0.43 d	2.01 ± 0.02 bc	1.87 ± 0.01 c
	25	15.71 ± 1.87 fg	6.55 ± 0.18 h	43.14 ± 0.04 a	84.89 ± 0.55 b	2.14 ± 0.02 a	2.03 ± 0.01 b
SIPF	5	21.90 ± 1.50 abc	9.33 ± 0.06 c	35.14 ± 0.20 h	68.23 ± 0.04 jk	1.74 ± 0.01 g	1.56 ± 0.01 h
	10	23.60 ± 0.92 a	10.65 ± 0.02 a	33.68 ± 0.07 i	65.75 ± 0.26 l	1.48 ± 0.01 i	1.15 ± 0.00 j
	15	20.30 ± 0.84 abcd	9.20 ± 0.11 cd	35.82 ± 0.19 g	69.28 ± 0.02 ij	1.78 ± 0.01 f	1.69 ± 0.01 g
	20	18.28 ± 1.91 cdefg	8.60 ± 0.30 e	39.39 ± 0.19 d	71.72 ± 0.58 fg	1.89 ± 0.01 d	1.79 ± 0.01 de
	25	17.31 ± 1.42 defg	7.41 ± 0.22 g	40.77 ± 0.29 b	76.19 ± 0.06 de	1.98 ± 0.02 c	1.81 ± 0.00 d
AIPF	5	22.44 ± 1.26 ab	10.13 ± 0.13 b	34.20 ± 0.06 i	67.79 ± 0.07 k	1.61 ± 0.01 h	1.53 ± 0.02 i
	10	19.83 ± 0.47 bcd	9.04 ± 0.13 cde	37.28 ± 0.21 f	70.04 ± 0.34 hi	1.79 ± 0.01 ef	1.75 ± 0.00 f
	15	17.36 ± 0.63 defg	7.90 ± 0.04 f	40.08 ± 0.36 c	75.38 ± 0.60 e	1.92 ± 0.01 d	1.80 ± 0.01 d
	20	16.15 ± 0.79 efg	6.65 ± 0.12 h	41.21 ± 0.15 b	81.50 ± 0.86 c	2.03 ± 0.02 b	2.00 ± 0.00 b
	25	15.16 ± 1.19 g	5.22 ± 0.01 i	43.49 ± 0.20 a	88.57 ± 0.33 a	2.15 ± 0.02 a	2.48 ± 0.01 a

Note: Values are expressed as mean ± SD (n = 3). Mean values within a column followed by different letters are significantly different according to Tukey’s test (*p* < 0.05). PF, pile fermentation; SDCLs, sun-dried coffee leaves; SPPF, spontaneous pile fermentation; SIPF, *S. cerevisiae*-inoculated pile fermentation; AIPF, *A. niger*-inoculated pile fermentation; TPC, total phenolic content; TFC, total flavonoid content. Trolox and acarbose as positive controls.

**Table 4:** Sensory attributes of coffee leaf dark tea.

Sample	PF Days	Appearance (25%)		Liquor Color (10 %)		Aroma (25 %)		Taste (30 %)		Infused Leaves (10 %)		Total Score
		Properties	Score	Properties	Score	Properties	Score	Properties	Score	Properties	**Score**	
SDCL	0	Light brownish-yellow; hairchested	81.67	Pale yellow	73.28	Fresh aroma	75.67	Bitter, astringent	71.11	Light yellowish-green; fully unfolded	74.33	75.01
SPPF	5	Dark brownish-yellow; thick	78.22	Light yellow-gold	72.33	Mellow	75.22	Fresh, brisk	75.67	Pale brown; mostly opened up	77.22	75.72
	10	Dark brown; thick	85.78	Yellow-orange	72.00	Fruity and woody aroma	74.11	Slightly bitter, slightly astringent	72.33	Light to medium brown; not fully flat	80.11	76.19
	15	Dark brown with hints of black; thick	80.22	Amber	81.11	Ripe fruity and woody aroma	80.00	Mellow and smooth; after-sweetness	77.56	Deep brown; partially unfolded	76.67	79.14
	20	Blackish brown; thick	78.22	Reddish brown	73.11	Woody aroma	78.22	Rich and full-bodied	74.33	Dark brown; curled	75.44	76.01
	25	Very dark brown to black; tight	82.11	Dark brown	72.33	Stale woody aroma	74.33	Thin	74.22	Black; tightly curled	77.11	75.83
SIPF	5	Dark brownish-yellow; thick	81.00	Light yellow-gold	78.00	Floral fruity	77.22	Slightly bitter; slightly astringent	74.44	Pale brown; mostly opened up	83.56	77.89
	10	Dark brown; tight	88.44	Yellow-orange bright	84.78	Ripe floral fruity	83.33	Fresh and rich; after-sweetness	83.56	Light to medium brown; not fully flat	78.44	84.15
	15	Dark brown with hints of black; tight	79.11	Amber	82.33	Ripe fruity	81.11	Rich; after-sweetness	81.67	Deep brown; partially unfolded	77.22	80.67
	20	Blackish-brown; tight	79.89	Reddish brown	82.11	Qu	80.11	Rich	80.44	Dark brown; curled	78.44	80.30
	25	Very dark brown to black; tight	86.44	Dark brown	73.44	Stale-Qu	79.89	Smooth	77.33	Black; tightly curled	83.44	79.82
AIPF	5	Dark brownish-yellow; tight	79.33	Light yellow-gold	78.56	Fruity and woody aroma	80.33	After-sweetness	71.11	Pale brown; not fully flat	75.67	80.00
	10	Dark brown; tight	88.11	Yellow-orange bright	86.89	Ripe fruity and woody aroma	85.00	Mellow and smooth	75.67	Light to medium brown; partially unfolded	89.00	85.97
	15	Dark brown with hints of black; tight	85.00	Amber bright	90.33	Fruity and woody aroma	84.56	Pure and mellow	72.33	Deep brown; partially unfolded	87.56	86.24
	20	Blackish-brown; tight	86.89	Amber	84.67	Floral fruity	83.67	Rich	77.56	Dark brown; curled	84.11	84.27
	25	Very dark brown to black; tight	81.33	Amber	76.67	Stale woody aroma	81.44	Smooth	74.33	Dark brown; curled	76.78	79.67

Note: PF, pile fermentation; SDCLs, sun-dried coffee leaves; SPPF, spontaneous pile fermentation; SIPF, *S. cerevisiae*-inoculated pile fermentation; AIPF, *A. niger*-inoculated pile fermentation.

## Data Availability

The original contributions presented in this study are included in the article. Further inquiries can be directed to the corresponding authors.
